# Burkholderia cepacia septicemia in a patient with acute myeloid leukemia in postchemotherapy bone marrow aplasia

**DOI:** 10.12669/pjms.295.3485

**Published:** 2013

**Authors:** Romeo-Gabriel Mihaila, Lucian Blaga

**Affiliations:** 1 Romeo-Gabriel Mihaila, MD, PhD, University of Sibiu, Faculty of Medicine, Str Lucian Blaga, nr 2A, Sibiu, 550169 Sibiu, Romania.; 2 Lucian Blaga, University of Sibiu, Faculty of Medicine, Str Lucian Blaga, nr 2A, Sibiu, 550169 Sibiu, Romania.

**Keywords:** Acute myeloid leukemia, Burkholderia cepacia, Ceftazidime, Cotrimoxazole

## Abstract

The patients with hematologic malignancies are predisposed to develop infections with unusual bacteria, like Burkholderia cepacia, which is frequently resistant to many antibiotics and antiseptics. We present the case of a female patient with acute myeloid leukemia type 2 on the background of myelodysplastic syndrome, from whom Burkholderia cepacia was isolated in blood culture, after the 2^nd^ cycle of induction. She was sensitive to ceftazidime, but its eradication was not easy. Five other patients were contaminated with this bacteria, but all of them had favourable evolution. The case is discussed in the context of those similar in literature.

## INTRODUCTION

Patients with malignant hemopathies are predisposed to develop various infections, including those with opportunistic germs, because they are immunosupressed. This immunosuppression is both disease- and chemotherapy-related. Sometimes, microorganism identification is a surprise for clinician due to its rarity or aggressiveness. Even if the practitioner has a huge experience he can find a bacteria that he has never met before. Bulkholderia cepacia is an example.

## CASE PRESENTATION

A female patient aged 60 was admitted to investigate a macrocytic anemia. Then she was diagnosed with acute myeloid leukemia type 2, on the background of myelodysplastic syndrome. She presented 37% peroxidazo-positive blasts in bone marrow; the flowcytometry established the following phenotype: CD34 +, CD33 +, HLA-DR-, CD61-, CD41-, CD14-, CD235-, CD19-, CD22-, CD10-, CD20-, CD8 -, CD4-, CD5 +, CD3ic-, MPOic +, CD22-; the cytogenetic examination found the presence of monosomy 8, and molecular biology tests have shown the absence of AML 1-ETO, FLT3-ITD, NPM, PML-RARa mutations. Because after a first cycle of induction with idarubicin + cytarabine (3+7) she had 25-38% peroxidazo-positive myeloblasts in bone marrow, she received a second cycle of induction of the same type. During aplasia, although she had been receiving intestinal decontamination treatment with rifaximin, acyclovir and cotrimoxazole, she had presented an episode of chills and fever. Burkholderia cepacia resistant to carbapenems but sensitive to ceftazidime and cotrimoxazole (with which it was treated) had been isolated in blood culture.

After a week without fever, another febrile episode occured, during which Burkholderia cepacia was isolated from central venous catheter, which was removed immediately. The fever disappeared quickly. She continued with ceftazidime until the erythematous papule at the site of implantation of catheter in the jugular vein disappeared. At the exit from aplasia she presented only 4% of blasts in the bone marrow (she was in complete remission). When she returned from her home, before the first cycle of consolidation it was isolated once more from sputum Burkholderia cepacia, which disappeared after ceftazidime treatment and it wasn’t more detected in the following two courses of consolidation polychemotherapy with idarubicin + high-dose cytarabine that followed and the disease has remained in complete remission. We note that after bacteria isolation at the patient, other five patients with hematologic malignancies during chemotherapy and bone marrow aplasia have been infected with Burkholderia cepacia, sensitive to ceftazidim and cotrimoxazole, which have evolved favourably under treatment. There were no deaths.

## DISCUSSION

In our hospital Burkholderia cepacia was not present for years. Characteristics of infection caused by this bacteria make it to be reckoned as redoubtable in services with immunosuppressed patients, through its tendency to produce nosocomial infections, therapeutic difficulties, and resistance to disinfection. The fact that it was resistant to carbapenems didn’t indicate necessarily that its presence to the presented patient was a nosocomial infection, because bacteria often live in the soil, where there are natural antibiotics, that it is often resistant to multiple antibiotics. It is possible that the period of aplasia could have favorized its replication and latent infection could become clinically manifest. The patient lives in rural areas and has often been exposed to contact with earth and plant products, including roots. The development of nosocomial infection from this patient has been possible because bacteria persists in aqueous medium, is found sometimes in cosmetics (moisturizing body milk), even of patients from Intensive Care Unit^1^, and even in antiseptics (it is resistant to chlorinated products, like chlorhexidine, cetylpyridinium chloride, etc.).^2^

Disinfection is difficult to do because antiseptics are not effective. Only careful patient isolation and washing hands with water after each contact with her allowed infection eradication. Burkholderia cepacia may be useful for environment: it enhances the degradation of organic compounds (e.g., it can increase the tolerance to toluene of yellow lupine plant and diminishes phytovolatilization of toluene into the atmosphere).^3^ Burkholderia cepacia colonizes and infects often patients with cystic fibrosis or chronic granulomatous disease but is also seen in cases of immunosuppression, like hematologic malignancies and solid tumors. In the lasts, Klebsiella pneumoniae and Escherichia coli are the most common gram-negative isolated bacteria, while Burkholderia cepacia is rare (9.5% of the group of less frequent gram-negative bacteria found in a study on hospitalized patients in Egypt, where it has been found in sputum, put, throat swab and bronchoalveolar lavage).^4^

More than 20 years ago, when it still called Pseudomonas cepacia, it was isolated from 22 patients hospitalized in an internal medicine clinic, in a year; all of them had neoplasms and most - hematologic malignancies (especially leukemias), more than half had complicated pneumonia; in decreasing order, the bacteria was sensitive to minocycline, ceftazidime, and ofloxacin.^5^ In a retrospective cross-sectional study recently published, 9 patients with hematologic malignancies have been infected with this bacteria. None had central venous catheter, and the source of infection could not be established. They didn’t respond quickly to antibiotics, and 9 of them died. Acute myeloid leukemia was the main risk factor, and the duration of infection was associated with neutropenia.^6^

Establishing the bacteriological diagnosis is not always easy, because there are at least 17 separate species. Identification of bacteria from 6 isolated harvested from a patient with acute myeloid leukemia and postchemotherapy neutropenia was difficult and required cellular fatty acid analysis and genomic typing realized by random amplified polymorphic DNA.^7^ Bacterial cell surface contains a lypopolysacharide with epitope variation between and within species. This is why it was prepared a specific monoclonal antibody against a lypopolysacharide, who produced 'ladder pattern' to some Burkholderia cepacia stains.^8^

Colonization and persistence of bacteria despite antibiotic treatment has other explanations, too: it has a high genomic instability and its fagocytosis induces caspase dependent neutrophils apoptosis, process in which oxygen free radicals are involved, which activate caspases.^9^ Because Burkholderia cepacia is frequently resistant to antibiotics, antisense technologies have been used to prepare phosphorodiamidate morpholino oligomers, to target the acpP gene, preventing the synthesis of a protein carrier, which is essential for growth. These oligomers have been bactericidal against this bacteria both in vitro and in animal studies.^10^

## CONCLUSION

Sometimes, the diagnosis of infection with Burkholderia cepacia is difficult, and at its establishing more immunosupressed patients can already be contaminated. Its resistance to antibiotics and antiseptics, and suppression of the host immune response are reasons that make it redoubtable.
